# Common Bile Duct Dilatation With Stones Indicates Requirement for Early Drainage in Patients With or Without Cholangitis

**DOI:** 10.4021/gr587w

**Published:** 2014-01-15

**Authors:** Yasunobu Yamashita, Kazuki Ueda, Hiroko Abe, Takashi Tamura, Masahiro Itonaga, Takeichi Yoshida, Hiroki Maeda, Takao Maekita, Mikitaka Iguchi, Hideyuki Tamai, Masao Ichinose, Jun Kato

**Affiliations:** a Second Department of Internal Medicine, Wakayama Medical University, 811-1, Kimiidera, Wakayama city, Wakayama 641-0012, Japan

**Keywords:** Common bile duct stones, Acute cholangitis, Biliary drainage

## Abstract

**Background:**

Some patients with common bile duct (CBD) stones develop cholangitis requiring drainage, while others do not. The aims of this study were to elucidate the clinical differences among patients with CBD stones who required and did not require emergent drainage, and to identify risk factors for the development of cholangitis requiring emergent drainage in patients with silent CBD stones.

**Methods:**

Clinical characteristics of consecutive patients with CBD stones who underwent endoscopic removal of stones or biliary drainage were analyzed retrospectively.

**Results:**

Of 101 patients analyzed, 32 had moderate or severe cholangitis as the indication for emergent drainage, and the remaining 69 did not. Patients who required emergent drainage were more likely to have gallstones (P = 0.029), dilated CBD (> 10 mm) (P = 0.004) and larger CBD stones (P = 0.019). By multivariate analysis, CBD dilation was the only significant differentiating clinical characteristic of the patients who required emergent drainage (OR = 3.75, 95% CI: 1.41-9.96, P = 0.008). Of the 35 patients with silent bile duct stones, eight required emergent endoscopic drainage during the waiting period. CBD dilation was also the only significant risk factor for the development of moderate or severe cholangitis among patients with silent bile duct stones (OR = 10.18, 95% CI: 1.09-94.73, P = 0.042).

**Conclusions:**

Dilated CBD (> 10 mm) was the only risk factor identified for requirement of early drainage in patients with CBD stones. Those who have silent CBD stones with CBD dilatation should undergo early drainage.

## Introduction

Acute cholangitis ranges from mild forms that respond to medical therapy to severe forms that lead to septicemia, a potentially lethal condition requiring emergent drainage of the bile duct [1, 2]. The major cause of acute cholangitis is presence of common bile duct (CBD) stones. It has been reported in the United States that approximately 70% of patients with acute cholangitis are able to achieve improvement with medical therapy alone [3]. However, the remaining cases do not respond to medical treatment and the clinical manifestations and laboratory data do not improve. Such cases may progress to sepsis with or without organ dysfunction and require appropriate management that includes intensive care, organ-supportive care and emergent biliary drainage, in addition to medical treatment. It has also been reported that the mortality rate due to acute cholangitis was up to approximately 10% despite appropriate antimicrobial therapy and biliary drainage [4, 5].

Endoscopic biliary drainage is an established mode of treatment for acute cholangitis, having high success rates and low morbidity and mortality [6-8]. Recent advances in and utilization of endoscopic biliary tract drainage along with the administration of antimicrobial agents have contributed to a decrease in the number of deaths due to acute cholangitis. However, it remains a life-threatening disease unless biliary tract drainage is performed in a timely manner.

In this context, the Tokyo Guideline 2013 for management of acute cholangitis and cholecystitis (TG13) indicated that patients with moderate or severe cholangitis, which was defined clearly in the guideline, required emergency or early biliary drainage [9]. However, to our knowledge, the factors contributing to occurrence of moderate or severe cholangitis requiring emergent drainage among patients with bile duct stones have not previously been reported. Particularly problematic in the clinical settings is that some patients with silent CBD stones develop acute cholangitis requiring emergent drainage before elective removal of stones by endoscopic retrograde cholangiopancreatography (ERCP).

Therefore, understanding differences between patients with CBD stones who require and do not require emergent drainage and identifying factors that contribute to occurrence of cholangitis requiring emergent drainage among patients with silent bile duct stones may be helpful for determining appropriate management strategies.

In this study, clinical characteristics of consecutive patients with CBD stones treated in a single tertiary center who required and did not require emergent drainage were compared retrospectively. Moreover, the risk factors among patients with silent CBD stones for the development of cholangitis requiring emergent drainage were identified.

## Materials and Methods

### Patients

**Figure 1:**
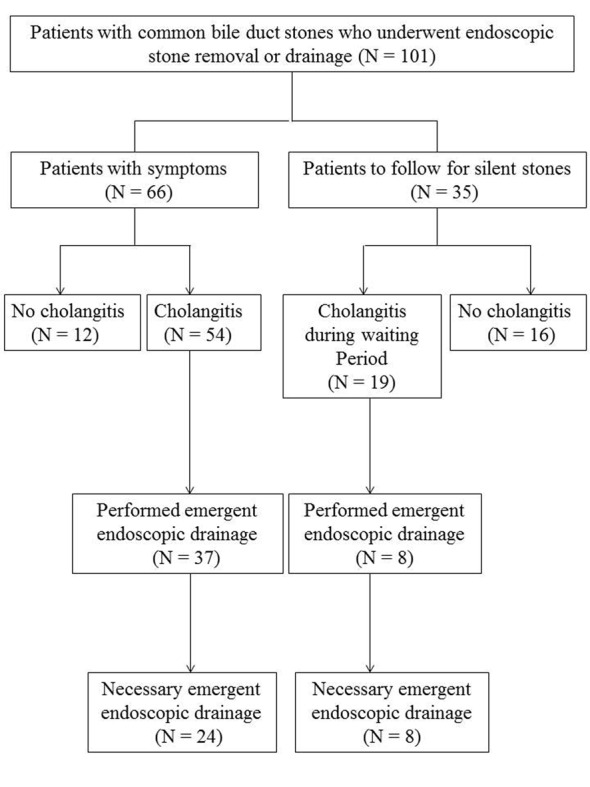
Study flow chart for treatment of patients with common bile duct stones.

Between December 2010 and December 2012, 101 consecutive patients with CBD stones underwent ERCP to remove stones or perform biliary drainage at Wakayama Medical University Hospital. The patients included those with and without cholangitis at the time of the endoscopic procedure. In addition, the endoscopic procedure for those patients was performed at various timings such as emergently at the first visit with serious cholangitis, electively due to silent stones and emergently during the waiting period for an elective procedure due to occurrence of cholangitis. The status of the patients and the timings of the endoscopic procedures are summarized in Figure 1.

The diagnosis of and severity assessment criteria for acute cholangitis were based on the TG13 (Table 1, 2) [9]. Before ERCP, all patients were evaluated hematologically with complete blood counts, prothrombin time, serum liver enzymes, C-reactive protein, serum amylase and serum renal function tests. Abdominal ultrasonography and computed tomography were also performed. The maximum diameter of the CBD was measured based on computed tomography images, and was considered dilated if it exceeded 10 mm. In patients with silent CBD stones and/or undergoing an elective procedure, CBD size was measured at the time of the first consultation and just before the endoscopic procedure, and changes in CBD size during that period were determined.

**Table 1:** Diagnostic Criteria for Acute Cholangitis

A	Systemic inflammation
A-1	Fever and/or shaking chills
A-2	Laboratory data: evidence of inflammatory response
B	Cholestasis
B-1	Jaundice
B-2	Laboratory data: evidence of abnormal liver function tests
C	Imaging
C-1	Biliary dilation
C-2	Evidence of the etiology on imaging

Definite diagnosis: one item in A, one item in B and one item in C.

**Table 2:** Severity Assessment Criteria for Acute Cholangitis

**Grade III (severe) acute cholangitis**
Grade III acute cholangitis is defined as acute cholangitis that is associated with the onset of dysfunction in at least one of any of the following organs/systems:
1. Cardiovascular dysfunction: hypotension requiring dopamine ≥ 5 µg/kg/min, or any dose of norepinephrine
2. Neurologic dysfunction: disturbance of consciousness
3. Respiratory dysfunction: PaO_2_/FiO_2_ ratio < 300
4. Renal dysfunction: oliguria, serum creatine > 2.0 mg/dL
5. Hepatic dysfunction: PT-INR > 1.5
6. Hematologic dysfunction: platelet count < 100,000/mm^3^
**Grade II (moderate) acute cholangitis**
Grade II acute cholangitis is associated with any two of following conditions:
1. Abnormal WBC count (> 12,000/mm^3^, < 4,000/mm^3^)
2. High fever (≥ 39 °C)
3. Age (≥ 75 years old)
4. Hyperbilirubinemia (total bilirubin ≥ 5 mg/dL)
5. Hypoalubuminemia (< STD x 0.7)
**Grade I (mild) acute cholangitis**
Grade I acute cholangitis does not meet the criteria of Grade III or Grade II acute cholangitis at initial diagnosis.

STD, lower limit of normal value.

### Endoscopic management of cholangitis

Informed consent was obtained from all patients or families before the endoscopic procedure was performed. This study was conducted in accordance with the Helsinki Declaration. All ERCP procedures were performed by experienced endoscopists. Selection of treatment procedure (stone removal, biliary stent placement, or nasobiliary tube) during emergent ERCP was made according to the individual endoscopist’s choice. For stone removal, endoscopic sphincterotomy (EST) with a pull-type sphincterotome was performed if EST had not been done previously. Multiple endoscopic procedures were performed if one procedure taking more than 30 min did not succeed in complete stone removal. Stones were removed using a basket and/or a balloon extraction catheter. A 7-Fr pigtail-tipped biliary stent was inserted without the challenge of stone removal if the patient’s vital signs were not stable because of advanced age and/or comorbidity.

### Data collection and statistical analysis

To identify differences between patients with CBD stones who required and did not require emergent endoscopic management, the following clinical factors were analyzed: gender, smoking status, alcohol consumption, diabetes, presence of gallstones, CBD dilation, size and number of CBD stones, history of EST and presence of periampullary diverticlum.

To identify risk factors for the development of severe or moderate cholangitis in patients with silent CBD stones, the following factors at the first consultation in our hospital were analyzed: gender, smoking status, alcohol consumption, diabetes, presence of gallstones, CBD dilation, size and number of CBD stones, total bilirubin, elevation of AST or ALT and evaluation of ALP or γ-GTP. These predictors were assessed using the χ^2^ test or Fisher’s exact test, as appropriate. The change of CBD size between silent CBD stone patient groups that required and did not require emergent drainage was assessed using the Mann-Whitney U test. P values less than 0.05 were considered statistically significant for all analyses. Predictive factors with P < 0.20 by univariate analysis were included in multivariate analysis using a backward stepwise logistic regression mode. All statistical analyses were performed with SPSS software (ver. 11) (SPSS, Chicago, IL, USA).

## Results

### Patients

A total of 101 patients (55 men and 46 women) with CBD stones who underwent ERCP were analyzed in this study. The mean age was 72 ± 15 years (range, 20 - 98 years). Of these, 66 patients had symptoms correlated with stones at presentation; 54 of these were diagnosed as acute cholangitis and 37 underwent emergent drainage. The other 35 patients had silent stones and had been followed while waiting for a scheduled endoscopic procedure; however, 19 of these developed cholangitis during the waiting period, and 8 of those underwent emergent drainage (Fig. 1).

All but one patient underwent an ERCP procedure under conscious sedation. One patient required artificial respiration under general anesthesia because of pulmonary insufficiency due to septic shock. One patient died from uncontrolled septicemia despite successful decompression of the biliary obstruction. All of the other patients with severe or moderate cholangitis rapidly improved after biliary decompression by ERCP. At the initial procedure, 65 patients underwent stone removal with or without residual stones, while the remaining 36 received biliary drainage only. During one or two additional procedures, 96 patients underwent complete stone removal. The remaining five underwent placement of biliary stents only, as the vital signs were not stable because of advanced age and comorbidity.

### Differences in clinical characteristics between bile duct stone patient groups that required and did not require emergent drainage

We assessed differences in clinical characteristics among patients with CBD stones who required and did not require emergent drainage, analyzing records of all of the 101 consecutive patients enrolled. Of these, 45 (45%) underwent emergent endoscopic drainage according to the individual physician’s judgment. However, according to the TG13 severity assessment criteria, 32 of these 45 patients would have been diagnosed as needing emergent drainage because of severe (13) or moderate (19) cholangitis. Therefore, the comparison was performed between the 32 patients with moderate or severe cholangitis requiring emergent drainage and the remaining 69 patients (Table 3).

**Table 3:** Characteristics of Patients With CBD Stones Who Required and Did Not Require Emergent Drainage

N = 101	Emergent drainage required (N = 32)	No emergent drainage required (N = 69)	P value
Patient (M/F)	32 (18/14)	69 (37/32)	0.81
Smoking	16	28	0.37
Alcohol	13	29	0.89
Diabetes	9	13	0.29
Gallstone	14	46	0.029
CBD dilation	25	33	0.004
No. of CBD stones > 2	17	35	0.82
Size of CBD stones > 10 mm	17	20	0.019
History of EST	6	7	0.23
Periampullary diverticlum	16	22	0.58

CBD, common bile duct; EST, endoscopic sphincterotomy.

Patients who required emergent drainage were more likely to have gallstones (P = 0.029), dilated CBD (> 10 mm) (P = 0.004) and larger CBD stones (P = 0.019) than those who did not require emergent drainage. By multivariate analysis, CBD dilation was the only significant differentiating clinical characteristic of the patients who required emergent drainage (OR = 3.75, 95% CI: 1.41-9.96, P = 0.008) (Table 4).

**Table 4:** Multivariate Analysis of Risk Factors for Cholangitis Requiring Emergent Drainage

	OR (95% CI)	P value
CBD dilation	3.75 (1.41-9.96)	0.008
Gallstone	2.44 (0.997-5.952)	0.051
Size of CBD stones > 10 mm	1.58 (0.59-4.23)	0.36

CBD, common bile duct; OR, odds ration; CI, confidence interval.

### Risk factors for the development of moderate or severe cholangitis requiring emergent drainage among patients with silent bile duct stones

In clinical settings, patients with silent CBD stones usually undergo removal of stones electively. However, some patients develop moderate or severe cholangitis requiring emergent drainage during the waiting period. Therefore, we determined risk factors for the development of cholangitis requiring emergent drainage among patients with silent CBD stones. Of the 35 patients with silent bile duct stones in our cohort, eight required emergent endoscopic drainage due to moderate or severe cholangitis during the waiting period. The median time from presentation to occurrence of cholangitis requiring emergent drainage was 60 days (7 - 540 days). The median change of the CBD size between the first visit and the ERCP procedure among patients who required and did not require emergent drainage was +5 mm (-1 - +9 mm) and ± 0 mm (-6 - +3 mm), respectively (P = 0.005).

By univariate analysis, patients who developed cholangitis requiring emergent drainage were more likely to have dilated CBD (> 10 mm) at the first visit than were those who did not (P = 0.025) (Table 5). By multivariate analysis too, CBD dilation was the only significant risk factor for the development of moderate or severe cholangitis among patients with silent bile duct stones (OR = 10.18, 95% CI: 1.09-94.73, P = 0.042) (Table 6).

**Table 5:** Univariate Analysis of Risk Factors for Development of Severe or Moderate Cholangitis in Patients With Silent CBD Stones

N = 35	Emergent treatment (N = 8)	Scheduled treatment (N = 27)	P value
Patient (M/F)	8 (5/3)	27 (17/10)	0.65
Smoking	6	15	0.29
Alcohol	5	13	0.38
Diabetes	3	6	0.33
Gallstone	3	16	0.25
CBD dilation	7	11	0.025
No. of CBD stones > 2	6	12	0.13
Size of CBD stones > 10 mm	4	9	0.33
Elevation of AST or ALT	4	15	0.55
Elevation of ALP or γ-GTP	5	20	0.41

CBD, common bile duct.

**Table 6:** Multivariate Analysis of Risk Factors for Development of Severe or Moderate Cholangitis in Patients With Silent CBD Stones

	OR (95% CI)	P value
CBD dilation	10.18 (1.09-94.73)	0.042
No. of CBD stones > 2	3.89 (0.58-26.12)	0.13

CBD, common bile duct; OR, odds ration; CI, confidence interval.

## Discussion

Development of acute cholangitis requires stasis or obstruction of bile compounded by the presence of bacteria. The most common cause of cholangitis is infection of the CBD due to blockage by a stone [10]. Acute cholangitis is a fatal disorder unless adequate biliary drainage is performed in a timely manner. In this context, accurate diagnosis and evaluation of severity of cholangitis are very important in clinical settings. However, the classic Charcot’s triad (namely, fever, jaundice and right upper quadrant pain) occurs in only 50%-75% of patients with acute cholangitis [1, 10, 11].

The Tokyo Guideline 2007 (TG07) detailed the world’s first severity assessment for acute cholangitis established at the International Consensus Meeting in Tokyo in 2006 [12]. However, using the severity assessment criteria of the TG07, one could not distinguish moderate cases requiring early drainage from mild cases that could be managed with antimicrobial agents alone. Because of these limitations of using the TG07 in clinical practice, the TG13 was made in order to improve severity assessment strategies at diagnosis to allow for provision of immediate infection source control in patients with acute cholangitis. However, while the criteria for moderate cholangitis in TG13 are consisted of patient age, fever and several blood makers, neither habitual factors such as smoking and drinking nor pathogenic factors of the hepatobiliary system including characteristics of stones and bile ducts that could affect the severity of cholangitis were included, since their effects are not as yet fully known.

In this study, we first analyzed clinical factors of patients with bile duct stones who underwent ERCP with or without moderate or severe cholangitis and showed that patients with cholangitis requiring emergent drainage were more likely to have dilated CBD (> 10 mm) than those who did not require it. Moreover, we demonstrated that the only risk factor analyzed in this study for the development of cholangitis requiring early drainage in patients with silent bile duct stones was also CBD dilatation. These findings would be helpful for identification of patients with silent CBD stones who are likely to develop moderate or severe cholangitis so they can be treated by ERCP in a timely fashion, resulting in reduction of morbidity and mortality due to severe cholangitis.

Previously, there have been two reports concerning risk factors for the development of acute suppurative cholangitis (ASC) with CBD stones [13, 14]. The ASC diagnosis corresponds with severe cholangitis in the TG13. The reported risk factors identified were advanced age, presence of gallstones, current smoking, periampullary diverticlum, impacted bile duct stones and neurologic disease. However, the only factor that both reports identified was advanced age. Advanced age as a risk factor was excluded from our analysis, because it is one of the diagnostic factors for moderate cholangitis in the TG13. Moreover, identification of risk factors for moderate or severe cholangitis appeared to be more important than identification of risk factors for ASC for use in clinical settings, because ASC is a life-threatening status that should be avoided at all costs. In this sense, our analysis and identification of one factor, CBD dilatation, for the development of moderate or severe cholangitis is unique and should prove to be very helpful in clinical practice.

Multivariate analysis of data in this study identified CBD dilation as the risk factor for cholangitis requiring emergent endoscopic drainage. A previous study indicated that the diameter and intraluminal pressure of the CBD in patients with acute calculous suppurative cholangitis were significantly greater than in patients with acute calculous non-suppurative cholangitis [15]. The current results seem to be consistent with results of that previous study. Presence of a dilated CBD with stones reflects high intraluminal CBD pressure, a condition that would render the patient susceptible to development of retrograde infections likely to induce more severe cholangitis.

A major limitation of this study is the retrospective design and the fact that criteria for performing emergent ERCP were not specified in advance. However, the study design did not appear to significantly affect the results, because we re-analyzed the data for the necessity of performing emergent ERCP according to TG13 criteria.

In conclusion, patients with cholangitis requiring emergent drainage due to bile duct stones were more likely to have a dilated CBD (> 10 mm) than those who did not require emergent drainage. Moreover, CBD dilation was the single significant risk factor identified in this study for the development of moderate or severe cholangitis in patients with silent CBD stones. On the basis of these results, we strongly recommended that patients with CBD stones accompanied by CBD dilation of > 10 mm in diameter undergo early removal of stones even when there are no cholangitis symptoms.
